# Sublingual Tropicamide Eye Drops for the Management of Clozapine-Induced Hypersalivation: A Case Series of Seven Patients

**DOI:** 10.3390/pharmacy14040101

**Published:** 2026-07-04

**Authors:** Seshadri Sekhar Chatterjee, Soumitra Das, Adesh Agrawal

**Affiliations:** 1 Department of Psychiatry, Central Queensland Hospital and Health Service (CQHHS), Rockhampton, QLD 4700, Australia; s.chatterjee@uq.edu.au; 2 School of Medicine, The University of Queensland, Rockhampton, QLD 4700, Australia; 3 Department of Psychiatry, Western Health, Melbourne, VIC 3021, Australia; 4 Department of Psychiatry, All India Institute of Medical Sciences (AIIMS), Deoghar 814152, Jharkhand, India; adesh.psychiatry@aiimsdeoghar.edu.in

**Keywords:** clozapine, hypersalivation, sialorrhea, tropicamide, treatment-resistant schizophrenia, anticholinergic, case series

## Abstract

Clozapine-induced hypersalivation (CIH) affects 30–80% of patients on clozapine and is a major contributor of non-adherence. Current managements, largely based on systemic anticholinergics, can worsen other clozapine-related side effects. Here sublingual tropicamide 1% eye drops can be a low-cost alternative worth evaluating. We report a case series of seven patients with schizophrenia treated with sublingual tropicamide 1% ophthalmic solution (4–8 drops/day) for CIH. Treatment response was assessed by patient-reported percentage reduction in salivation. All seven patients reported improvement (25–80%). One patient reported a transient bitter taste; no systemic anticholinergic effects occurred. Sublingual tropicamide was associated with patient-reported improvement in CIH and was well tolerated in this small, uncontrolled series. Its short duration of action and local administration may offer practical advantages over systemic anticholinergics, though the mechanism remains unproven. Randomised trials with validated outcome measures are needed to establish its efficacy and safety.

## 1. Introduction

Clozapine is the most effective antipsychotic for treatment-resistant schizophrenia (TRS), defined as inadequate response to at least two adequate antipsychotic trials [1]. Despite this, its adverse effect burden remains a major barrier to its use.

Clozapine-induced hypersalivation (CIH), or sialorrhea (CIS), affects 30–80% of treated patients and is one of the most distressing and stigmatising side effects of clozapine [2,3]. In a nationwide Australian cohort of over thousand patients, sialorrhea was the single most common self-reported adverse effect, occurring in 80.3% of participants [4]. CIH may begin from the first dose or emerge months later. It disrupts sleep, causes social embarrassment, carries an aspiration risk, and can lead to treatment discontinuation [5,6].

The pathophysiology of CIH is not fully understood and is likely multifactorial. Salivary secretion is generally attributed to M3 (and, to a lesser extent, M1) muscarinic receptors [7]. Clozapine blocks these receptors but has also been proposed to act as a partial agonist at M4 receptors, which could paradoxically stimulate salivation; however, M4 receptor distribution in human salivary tissue is far less established than that of M3, and this remains one hypothesis among several rather than a confirmed mechanism [2,6,7] Antagonism at alpha-2 adrenoceptors and impaired swallowing during sleep have also been proposed as contributing factors [5,6]. How much each mechanism contributes, and whether this varies between patients, is unknown.

Management of CIH lacks consensus and high-quality evidence [8]. Non-pharmacological measures—chewing gum, positional changes, swallowing exercises—are often insufficient. Pharmacological options include systemic anticholinergics (hyoscine, glycopyrrolate, sublingual atropine), alpha-2 agonists (clonidine), amisulpride, and, for refractory cases, botulinum toxin [9,10]. Systemic anticholinergics, however, risk worsening clozapine’s own gastrointestinal and urinary side effects, limiting their long-term use [8].

Sublingual delivery aims to act locally on salivary gland receptors while limiting systemic absorption, though this has rarely been directly measured. Sublingual atropine has shown efficacy in case reports [11,12] and a randomised controlled trial [13]. Its longer half-life and greater blood–brain barrier penetration relative to tropicamide have prompted interest in shorter-acting alternatives, though direct comparisons are lacking.

Tropicamide 1% ophthalmic solution is a short-acting, non-selective muscarinic antagonist (duration of action 6–8 h). A rodent study suggested a possible role for M4 antagonism in tropicamide’s effects on jaw movement [14], but this has not been confirmed in human salivary tissue. Lloret et al. (2011) [15] showed efficacy of intra-oral tropicamide for Parkinson’s disease sialorrhea in a randomised pilot trial. Kilic et al. (2017) [16] first reported sublingual tropicamide for CIH specifically. A later retrospective chart review by Mutlu et al. (2023) [17] found tropicamide used as a first-step agent in 49% of CIH cases, with a median 33% reduction in nocturnal wet pillow diameter and no serious adverse events. Tropicamide is also inexpensive and widely available in India, making it practically accessible off-label.

Repurposing tropicamide for CIH is therefore not a new idea, having been described in one case report and one chart review [16,17]. However, clinical descriptions remain scarce, and none have come from a South Asian setting. We report seven patients with schizophrenia or TRS given sublingual tropicamide for CIH, and discuss dosing strategies, response patterns, and what these observations add to the existing literature.

## 2. Case Descriptions

The cohort comprised five males and two females, mean age 36.3 years (range 21–54). Five had TRS and two had schizophrenia. Illness duration ranged from 5 to 24 years. CIH onset ranged from day 1 to about six months after starting clozapine, at doses of 50–500 mg/day. Table 1 summarises all cases; individual descriptions follow.

### 2.1. Case 1 (SRK): Hypersalivation from Day 1—Dose-Dependent Response

A 43-year-old male with seven years of TRS started clozapine and reported moderate-to-severe drooling on day 1, at 50 mg. Sublingual tropicamide 4 drops/day for four days gave 30% improvement; increasing to 6 drops/day for two weeks gave 60%. Tapering caused a brief, self-resolving rebound. No adverse effects occurred. This case shows that CIH can appear from the first dose, even at low clozapine doses, and that stepped dosing can produce progressive benefit.

### 2.2. Case 2 (AN): Response with a Short Course

A 54-year-old male with 24 years of schizophrenia, meeting TRS criteria after multiple failed antipsychotic trials, was switched to clozapine. He developed hypersalivation on day 13, at 75 mg. Tropicamide 4 drops/day for seven days gave an 80% reduction—one of the two largest responses in this series, alongside Case 4. He was tapered over three months with no relapse or adverse effects. This case suggests a brief, low-dose course may give a substantial, durable response in early CIH, though natural fluctuation during early titration cannot be excluded.

### 2.3. Case 3 (KA): Early-Onset CIH—Transient Bitter Taste as Side Effect

A 38-year-old female with 12 years of schizophrenia developed hypersalivation by day 7 of clozapine, at 50 mg. Tropicamide 4 drops/day for two days gave 50% improvement—an intermediate response similar to Cases 5 and 7. This patient had the only adverse effect in the series: a transient bitter taste that resolved without intervention.

### 2.4. Case 4 (ST): Sleep-Disrupting CIH—Response with Intermittent Dosing

A 32-year-old female with 12 years of illness, having failed amisulpride and olanzapine, met TRS criteria and started clozapine. By day 14, at 125 mg, she had hypersalivation disrupting her sleep. Her clinician chose an intermittent regimen—4 drops/day, four days/week, with drug-free weekends—based on response and tolerability rather than a fixed protocol. This gave 80% improvement with no adverse effects and was well accepted. This suggests that intermittent dosing may suit some patients, though this needs confirmation in controlled studies.

### 2.5. Case 5 (VS): Moderate-Dose Clozapine CIH—Improvement with Intermittent Dosing

A 41-year-old male with eight years of TRS developed hypersalivation on day 33, at 300 mg. Intermittent tropicamide (4 drops/day, four days/week for three weeks) gave 50% improvement with no adverse effects. Onset at day 33—after initial titration but before dose stabilisation—shows CIH can emerge at varying points in treatment.

### 2.6. Case 6 (KP): Late-Onset CIH at High Dose—Stepwise Escalation

A 25-year-old male with 11 years of TRS developed hypersalivation after about six months on clozapine, at 500 mg—the latest onset and highest dose in this series. Tropicamide 4 drops/day for three weeks gave 50% improvement; escalating to 6 drops/day for two to three months improved this to 70%, followed by gradual tapering. No adverse effects occurred. Escalation was based on the clinician’s judgement of inadequate response rather than pre-set criteria.

### 2.7. Case 7 (SK): Dose Escalation Required for Adequate Response

A 21-year-old male with five years of schizophrenia developed hypersalivation by day 19 of clozapine, at 100 mg. An initial intermittent course (4 drops/day, four days/week) gave only 25% improvement—the most modest initial response in this series. Escalating to 8 drops/day (four days/week) achieved 50%, similar to Cases 3 and 5. No adverse effects occurred. The reason for the weaker initial response is unclear and may reflect individual variability in mucosal absorption, though this was not directly assessed.

## 3. Discussion

This series describes sublingual tropicamide 1% for CIH in seven patients with schizophrenia or TRS—five males and two females, aged 21 to 54, with illness durations of 5 to 24 years. All seven reported improvement (25–80%), and no systemic anticholinergic effects occurred. The only adverse effect was a transient bitter taste in Case 3 (KA), which resolved spontaneously.

### 3.1. Mechanistic Considerations

The mechanism of CIH is discussed in the Introduction; we did not test it directly. No pharmacodynamic, receptor-binding, or plasma drug-level data were collected in this series, so our findings cannot confirm or refute the proposed M4 hypothesis and should not be read as evidence of tropicamide’s receptor selectivity in humans. Regardless of mechanism, tropicamide’s short action (6–8 h) may offer a practical advantage by allowing targeted nocturnal dosing without all-day dry mouth.

### 3.2. Route of Administration

Sublingual delivery is intended to limit systemic anticholinergic exposure, but we did not measure absorption or anticholinergic burden directly, so this should be read as an assumption rather than a finding. The same rationale underlies sublingual atropine, which showed efficacy in a randomised trial [13]. Tropicamide’s shorter half-life and lower blood–brain barrier penetration relative to atropine have been suggested as a possible tolerability advantage [11,12], but no head-to-head comparison exists. The single adverse effect in our series—a transient bitter taste—is consistent with local mucosal contact, but seven patients followed briefly cannot rule out rarer or longer-term effects, including cumulative anticholinergic burden.

### 3.3. Response Patterns

The patterns below are exploratory hypotheses, not conclusions, given the very small sample. Patients with earlier CIH onset and lower clozapine doses (Cases 1–4) tended to show higher reported improvement than the case with the latest onset and highest dose (Case 6: 500 mg, onset at six months), which needed escalation and the longest course to reach a comparable response. One possibility is that higher or longer clozapine exposure requires greater antimuscarinic effect to control symptoms, but we did not measure plasma levels, so this remains speculative. We saw no clear pattern by sex and have avoided drawing conclusions from only two female and five male patients. Three patients (Cases 4, 5, 7) used intermittent dosing (four days/week) and all achieved improvement (50–80%) comparable to daily dosing, suggesting this may be a feasible strategy—though it was not formally compared with daily dosing here and needs prospective testing.

### 3.4. Relationship to Existing Literature

Tropicamide for CIH was first reported by Kilic et al. (2017) [16] in a single case, then in a larger chart review by Mutlu et al. (2023) [17], who found it used as a first-step agent in 49% of CIH cases, with a median 33% reduction in wet pillow diameter and no serious adverse events. Our results are broadly consistent with this in terms of tolerability. This series adds observations not previously reported for tropicamide in CIH: onset timing relative to clozapine dose across 50–500 mg/day; individual variability in the need for dose escalation; and intermittent (four days/week) dosing in three patients. Given the small sample, these observations should inform—not replace—future trial design. Preclinical work [14] and a Parkinson’s disease trial [15] lend indirect pharmacological support but do not address CIH in humans directly.

### 3.5. Practical and Resource Considerations

**Figure 1:**
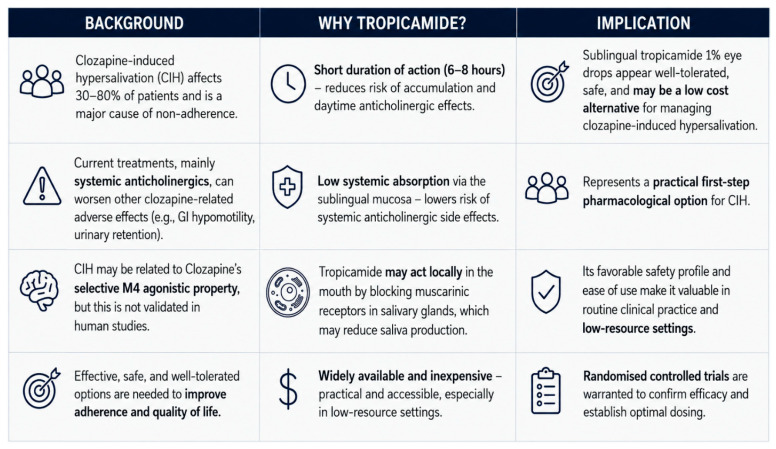
Graphical summary of background, rationale for tropicamide, and clinical implications.

Tropicamide eye drops are inexpensive, widely available in Indian pharmacies, and need no compounding—practical advantages in resource-limited settings. It has the potential to be a low-cost alternative, and a cost-effectiveness analysis may be beneficial. Systemic anticholinergics such as hyoscine and glycopyrrolate risk worsening clozapine’s own constipation and, rarely, ileus [8,18]. Botulinum toxin is effective in refractory cases but needs specialist administration and repeat injections, limiting access in many settings. Figure 1 summarises these considerations.

### 3.6. Limitations and Recommendations for Future Research

This series has several limitations. It is small (*n* = 7), uncontrolled, and unblinded. The primary outcome was a patient-reported percentage estimate, not a validated instrument such as the Drooling Severity and Frequency Scale (DSFS) or Nocturnal Hypersalivation Rating Scale (NHRS); no such tool was used because tropicamide was given as routine off-label care, not within a research protocol. We cannot exclude reporting bias or a placebo effect, given the lack of blinding. Dosing decisions—escalation, intermittent scheduling, tapering—were made clinically rather than by protocol, so any apparent dose–response relationship cannot be fully separated from this variability. No pharmacokinetic or plasma-level data were collected, and the proposed mechanism remains hypothetical. No comparator group was used, and short follow-up in several cases precludes conclusions about longer-term effects or anticholinergic burden. Future studies should use a prospective, ideally randomised, placebo-controlled design with validated rating scales, blinded assessment, standardised dosing with pre-specified escalation criteria, plasma-level monitoring, and direct comparison with sublingual atropine.

## 4. Conclusions

In this small, uncontrolled series, sublingual tropicamide 1% was associated with patient-reported reductions in CIH across seven diverse patients, with no systemic adverse effects. Its short action and local delivery may offer practical advantages over systemic anticholinergics, though the mechanism remains unconfirmed. The response patterns observed—varying by clozapine dose and CIH onset timing—are hypotheses to guide, not replace, future controlled research. Randomised, placebo-controlled trials with validated outcome measures, blinded assessment, standardised dosing, and plasma-level monitoring are needed before tropicamide’s efficacy, safety, and optimal dosing for CIH can be established.

## Figures and Tables

**Table 1:** Summary of clinical characteristics and treatment outcomes in seven patients.

Case No.	Age/Sex	Illness Duration	CIS Onset	Clozapine Dose at Onset	Tropicamide Dose & Regimen	Improvement/AE
**1 (SRK)**	43M	7 years	Day 1	50 mg	4 drops/day × 4 days; then 6 drops/day × 2 weeks; taper	30% → 60%; Nil
**2 (AN)**	54M	24 years	Day 13	75 mg	4 drops/day × 7 days; taper over 3 months	80%; Nil
**3 (KA)**	38F	12 years	Day 7	50 mg	4 drops/day × 2 days	50%; Bitter taste
**4 (ST)**	32F	12 years	Day 14	125 mg	4 drops/day, 4 days/week (drug-free weekends)	80%; Nil
**5 (VS)**	41M	8 years	Day 33	300 mg	4 drops/day, 4 days/week × 3 weeks	50%; Nil
**6 (KP)**	25M	11 years	~6 months	500 mg	4 drops/day × 3 weeks; 6 drops/day × 2–3 months; taper	50% → 70%; Nil
**7 (SK)**	21M	5 years	Day 19	100 mg	4 drops/day, 4 days/week; increased to 8 drops/day, 4 days/week	25% → 50%; Nil

CIS: Clozapine-induced sialorrhea; M: Male; F: Female; AE: Adverse effect.

## Data Availability

The data presented in this study are available on request from the corresponding author due to privacy.
